# Chemotherapy negatively impacts the tumor immune microenvironment in NSCLC: an analysis of pre- and post-treatment biopsies in the multi-center SAKK19/09 study

**DOI:** 10.1007/s00262-020-02688-4

**Published:** 2020-08-07

**Authors:** M. A. Amrein, E. D. Bührer, M. L. Amrein, Q. Li, S. Rothschild, C. Riether, R. Jaggi, S. Savic-Prince, L. Bubendorf, O. Gautschi, A. F. Ochsenbein

**Affiliations:** 1 Department for BioMedical Research, University of Bern, Bern, Switzerland; 2 Graduate School of Cellular and Biomedical Sciences, University of Bern, Bern, Switzerland; 3 Swiss Group for Clinical Cancer Research Coordinating Center, Bern, Switzerland; 4 Department of Medical Oncology, University Hospital Basel, Basel, Switzerland; 5 Institute of Pathology, University Hospital Basel, Basel, Switzerland; 6 Department of Medical Oncology, Cantonal Hospital Lucerne, Lucerne, Switzerland; 7 Department of Medical Oncology, Inselspital, Bern University Hospital, University of Bern, Bern, Switzerland

**Keywords:** Non-small-cell lung cancer, Immune microenvironment, Chemotherapy, Immune checkpoint

## Abstract

**Background:**

Over the past few years, immune checkpoint inhibitors have changed the therapeutic landscape of non-small-cell lung cancer (NSCLC). Response to immune checkpoint inhibitors correlates with a pre-existing anti-tumoral immune response. Checkpoint inhibitors have been introduced as second-line therapy and are only very recently used as monotherapy or in combination with chemotherapy as first-line treatment of NSCLC. However, the effect of conventional first-line platinum-based chemotherapy on the immune infiltrate in the tumor is largely unknown.

**Methods:**

We measured the gene expression of a custom set of 201 cancer- and immune-related genes in 100 NSCLC tumor biopsies collected before chemotherapy and 33 re-biopsies after platinum-based chemotherapy at the time point of progression. For 29 patients matched pre- and post-chemotherapy samples could be evaluated.

**Results:**

We identified a cluster of 47 co-expressed immune genes, including PDCD1 (PD1) and CD274 (PD-L1), along with three other co-expression clusters. Chemotherapy decreased the average gene expression of the immune cluster while no effect was observed on the other three cluster. Within this immune cluster, CTLA4, LAG3, TNFRSF18, CD80 and FOXP3 were found to be significantly decreased in patient-matched samples after chemotherapy.

**Conclusion:**

Our results suggest that conventional platinum-based chemotherapy negatively impacts the immune microenvironment at the time point of secondary progression.

**Electronic supplementary material:**

The online version of this article (10.1007/s00262-020-02688-4) contains supplementary material, which is available to authorized users.

## Introduction

For decades, treatment of non-small cell lung cancer (NSCLC) relied on combinations of platinum-based chemotherapy regimens with rather limited success. However, the emergence of immune checkpoint inhibitors is now rapidly changing the therapeutic landscape for NSCLC. In 2015, the anti-programmed cell death 1 (PD-1) antibodies nivolumab and pembrolizumab were the first immune checkpoint inhibitor to be approved by the food and drug administration (FDA) for the treatment of advanced squamous and non-squamous NSCLC [1–4]. Recently, two additional blocking antibodies against PD ligand 1 (PD-L1), atezolizumab and durvalumab, have been approved for the treatment of non-resectable advanced NSCLC [5, 6]. However, only a minor fraction of NSCLC patients has objective responses to these immunomodulatory drugs, yet these responses are often long-lasting. Reported response rates to nivolumab range between 15 and 33% for the treatment of squamous NSCLC [3, 4, 7] and between 12 and 19% for the treatment of non-squamous NSCLC [1, 4, 8]. For pembrolizumab, atezolizumab and durvalumab, similar response rates have been reported for either histological subtypes [2, 5, 9].

Since the approval of the anti-PD-1 and anti-PD-L1 antibodies, most NSCLC patients were treated with immune checkpoint inhibitors in second-line therapy upon tumor progression after standard platinum-based chemotherapy. However, the KEYNOTE-24 study documented that first-line treatment with pembrolizumab was superior to standard chemotherapy in a subgroup of patients with > 50% PD-L1 expression on the tumor cells [10]. It is especially noteworthy that the survival benefit was observed despite a high degree of crossover of patients from chemotherapy to pembrolizumab (43.7%) upon disease progression [10]. However, similar trials with nivolumab and durvalumab as first-line monotherapy failed to improve progression-free survival (PFS) [11, 12]. In contrast, the combination of nivolumab with the anti-CTLA-4 blocking antibody ipilimumab improved PFS in patients with high tumor mutational burden compared to standard chemotherapy [13].

It has become increasingly clear that response to immune checkpoint inhibitors correlates with the presence of a pre-existing anti-tumoral immune response. Already in the first trials, it became clear that the presence of tumor-infiltrating T cells (“hot tumors”) is a predictive marker for response to immune checkpoint inhibitor [14]. In subsequent studies, immune-related gene expression patterns, IFNγ signatures and Th1 cytokines have been identified as predictive factors for the treatment with PD1/PD-L1 blocking molecules [9, 15]. Therefore, we sought to analyze the effect of conventional chemotherapy on the expression of immune-related genes in NSCLC tumor samples. We measured the gene expression of 201 cancer- and immune-related genes in NSCLC tumors before conventional platinum-based chemotherapy and at the time point of progression. RNA was isolated from formalin-fixed paraffin-embedded (FFPE) tumor biopsies collected as part of the SAKK19/09 study from epidermal growth factor receptor (EGFR) wild-type, non-squamous NSCLC patients. No significant difference was found when pre- and post-therapy samples were compared at a single gene level. Therefore, using a bioinformatics approach, we built a weighted co-expression network to identify modules of co-expressed genes. Out of four identified co-expression modules, one module was enriched for genes related to adaptive immune responses. Overall, chemotherapy was found to reduce the average gene expression profile of this immune module. This suggests a negative impact of platinum based chemotherapy on anti-tumoral immunity.

## Methods

### Trial design and objectives

**Fig. 1:**
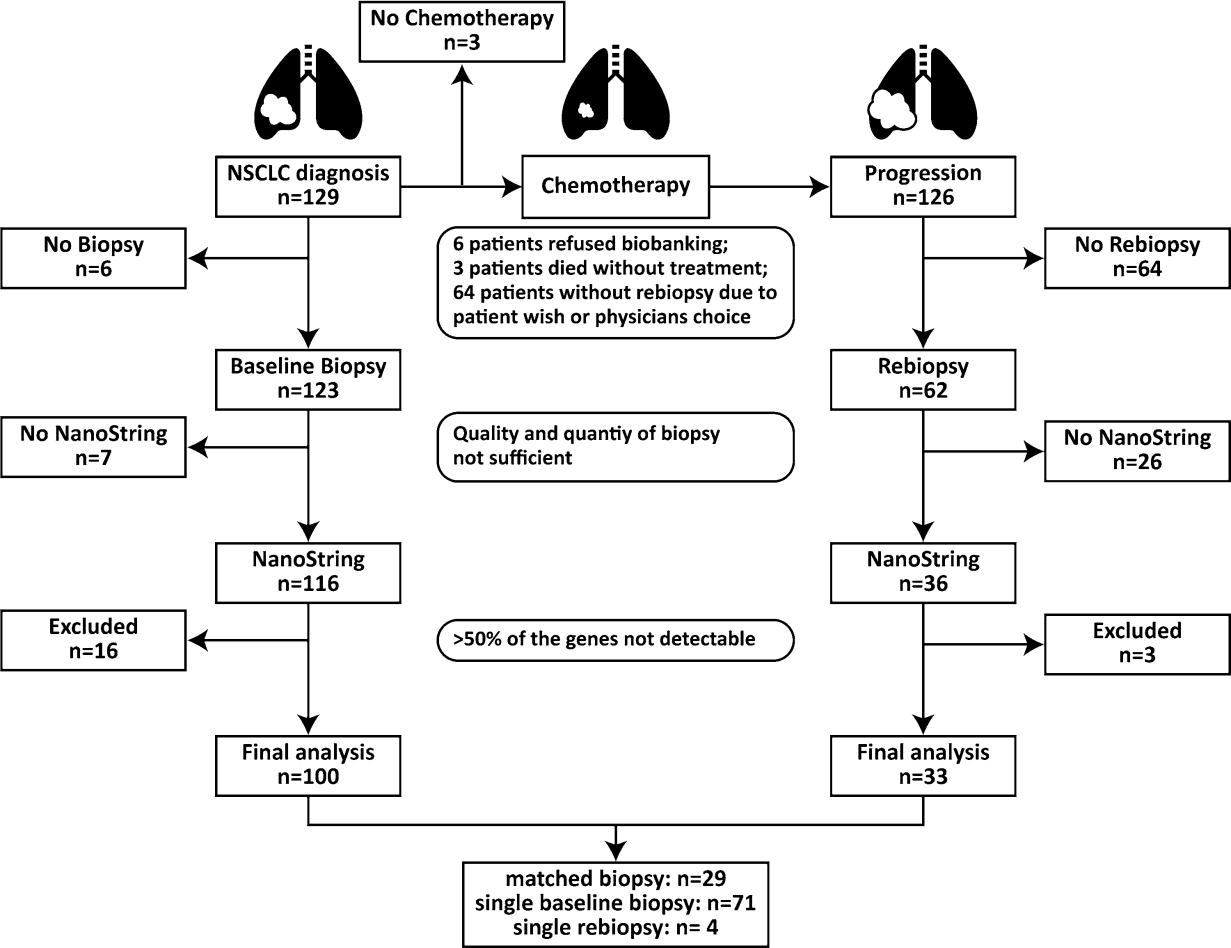
Flowchart diagram of the biopsy collection and analysis

The SAKK 19/09 clinical trial design has been previously described [16, 17]. Briefly, the trial was designed as a non-randomized multi-center phase II study. A total of 152 patients were enrolled. The inclusion criteria were defined as followed: histologic diagnosis of non-squamous NSCLC, stage M1a/b according to the 7th TNM edition, no brain metastasis according to computer tomography (CT) and no contradictions for the trial treatment. Patients receiving prior chemotherapy or molecular targeted therapy for metastatic disease, with the exception of neoadjuvant or adjuvant chemotherapy if terminated 6 months before registration, were excluded from the study. Overall, three patients were excluded from further analysis, as they did not receive any treatment (one died before treatment initiation, one had a persisting pilonidal sinus and one showed a decreased performance status). Patients were stratified according to their EGFR mutation status. Patients with EGFR mutations (Stratum A: del9 or L858R) (*n* = 20) were excluded and only EGFR wildtype patients (Stratum B) were included for further analysis (*n* = 129). Patients with EGFR wild type received either four cycles cisplatin (CIS) with bevacizumab (BEV) plus pemetrexed (PEM) (Cohort 1: *n* = 77) or were treated without BEV (Cohort 2: *n* = 52). The purpose of this clinical trial was to demonstrate that (1) tailored therapy according to EGFR mutation status was promising for further investigation and (2) to test whether BEV plus CIS/PEM was superior to CIS/PEM alone [16, 17]. In addition, the study required a tumor biopsy at the time point of progression after first-line therapy to analyse resistance mechanisms to chemotherapy and changes in the immune infiltrate in the tumor. In the present study, we analyzed the gene expression profiles from EGFR wild type tumors (Stratum B) at baseline and at the time point of progression after first-line treatment (Fig. 1).

### Treatment and follow-up protocol

Patients with EGFR wild type received four cycles of induction therapy with CIS 70 mg/m^2^ and PEM 500 mg/m^2^, with or without BEV 7.5 mg/m^2^ every 3 weeks. Patients without progression after four cycles were treated with maintenance therapy with PEM 500 mg/m^2^, with or without BEV 7.5 mg/m^2^ every 3 weeks until confirmed progression. If CIS-related toxicity > grade 2 was observed, CIS was substituted with carboplatin area under the curve 5. Thoracoabdominal CT scans were performed at baseline and follow-up scans were done every 6 weeks until confirmed progression. Response was evaluated according to Response Evaluation Criteria in Solid Tumors (RECIST) 1.1.

### Sample collection

Written informed consent was obtained for the longitudinal collection of formalin-fixed and paraffin-embedded (FFPE) tumor biopsies. Diagnostic tumor biopsies were collected at baseline and all patients agreed to repeat the tumor biopsy upon disease progression. All FFPE tumor biopsies were assessed by a board certified pathologist to evaluate adequacy for laser capture microdissection and gene expression analysis. The laser capture microdissection was performed on a Laser PALM Microlaser Technology System.

### mRNA analysis

Total RNA was extracted from microdissected tumor tissue using the FFPE Tissue RNA Extraction Kit from amsbio, (Bioggio-Lugano, Switzerland), according to the manufacturer’s protocol. RNA quantity was assessed on a Nanodrop and quality was assessed on a Bioanalyzer. Gene expression was analyzed on a NanoString nCounter platform (Nanostring Technologies). Briefly, 100 ng RNA were incubated overnight at 65 °C with Nanostring probe sets.

All samples were analyzed with a custom probe code set consisting of a panel of 201 genes.

The genes were selected from literature to evaluate the effect of the different treatment regimens on the expression immune inhibitory and activating ligands/receptors, NFkB- and WNT-signalling related genes, as well as genes involved in the nucleotide excision repair pathway and angiogenesis (Table S3). The code set contained internal positive (spiked RNA to assess overall assay performance) and negative controls (orphan probes for background estimation). Counts of hybridized probes were measured on a nCounter Analysis system and raw data was normalized using a homemade Excel macro as described previously [18]. Briefly, the negative control averages plus 2 SD were subtracted to correct for background noise and values below 0 were set to 1. Then, the geNorm method was used to select adequate genes for normalization from the included set of 6 candidates based on their relative stability [19]. For the final normalization, the geometric mean of the selected normalization genes was calculated and used as the normalization factor. Normalized expression values were log2 transformed and samples where > 50% of the probes were not detectable were removed from further analysis (Fig. 1). The gene expression data are deposited at the NCBI Gene Expression Omnibus (GEO) under the accession number GSE154286.

### Co-expression module detection (gene clusters)

The weighted gene co-expression network analysis (WGCNA) R package was used to identify clusters of co-expressed genes, referred to as modules. The WGCNA function 'blockwiseModules' was implemented to create a co-expression network and to extract modules from a weighted and signed correlation matrix [20, 21]. The blockwiseModules function was implemented using the following parameters: power = 8, minModuleSize = 10, neworkType = “signed”. Briefly, Pearson correlation coefficients were calculated for any two genes across all samples. Then, a weighted network matrix was obtained by transforming the Pearson correlation matrix with a power function. Next, the topological overlap measure (TOM) was calculated using a dynamic tree-cutting algorithm. 1-TOM was finally used as distance measure to cluster genes hierarchically and modules were determined from the resulting dendrogram by choosing a height cutoff 0.5. Gene expression profiles of the individual modules are summarized by the module eigengene (ME) expression value, defined as the first principle component of the expression matrix.

### Functional module annotation

To classify the identified co-expression modules functionally, gene ontology (GO) enrichment analysis was performed using ‘clusterProfiler’ in R [22]. Modules were searched for over-represented biological process (bp) GO terms. Enrichment analysis was performed against a background list containing all 201 genes. This ensures that only GO terms which are identified by the entire gene set (*n* = 201) and which are enriched in a specific module are detected. GO terms with a *p* value of *p* < 0.01 were considered as significantly enriched.

## Results

### Characteristics of the NSCLC dataset

We analyzed the expression of 201 genes related to apoptosis, angiogenesis, cell cycling, stemness and immunity in FFPE tumor biopsies from NSCLC patients collected before and after platinum-based chemotherapy at the time of progression. The samples analyzed in this study were collected as part of the SAKK19/09 study from EGFR wild type patients (Table 1) [16, 17]. Patients enrolled in the study were either treated with CIS + PEM + BEV (*n* = 77) or CIS + PEM (*n* = 52). Allocation to the treatment arms was not randomized. The CIS + PEM + BEV cohort was enrolled first, followed by the CIS + PEM cohort. As shown previously, the lack of randomization did not cause any obvious imbalances in the baseline characteristics between the two cohorts [17]. Most patients (87%) did not receive any kind of treatment prior to their enrolment. Previous treatments included surgery, radiotherapy or adjuvant chemotherapy.

**Table 1:** Patient characteristics

	Cohort (*n* = 129)	
	*n*	%
Gender		
F	56	43*.*4
M	73	56*.*6
Urine dipstick for proteinuria		
1+	22	17*.*1
Negative	107	82*.*9
Smoking		
Yes, currently	67	51*.*9
Yes, formerly	45	34*.*9
Never	17	13*.*2
Tumor stage		
M1a	22	17*.*1
M1b	107	82*.*9
Comorbidities		
No	29	22*.*5
Yes	100	77*.*5
Chemotherapy regimen		
CIS + PEM + BEV	77	59*.*7
CIS + PEM	52	40*.*3
Previous treatment		
No	113	87*.*5
Chemotherapy	8	6*.*2
Radiotherapy	4	3*.*1
Surgery	15	11*.*6
Written consent for biobanking		
Yes	123	95*.*3
No	6	4*.*7

All categories except “previous treatment” sum up to 100%. Patients may have received more than one previous treatment modality before inclusion into the study

*CIS* cisplatin, *PEM* pemetrexed, *BEV* bevacizumab

Baseline tumor biopsies were collected from 123 of 129 patients (95.3%; CIS + PEM + BEV *n* = 74; CIS + PEM *n* = 49) and matched tumor re-biopsies were collected at time point of progression from 62 patients (48.1%; CIS + PEM + BEV *n* = 36; CIS + PEM *n* = 26) (Fig. 1 and Table S1). The main reasons for not performing re-biopsies were patient-wish and the decision of the physician. The frequency of re-biopsies was comparable in the BEV + cohort and in the BEV-cohort (Table S1). We were able to analyze the gene expression on the Nanostring nCounter platform (NanoString Technologies) for 116 of 123 baseline samples and 36 of 62 re-biopsies (Fig. 1 and Table S1). Samples where the expression of more than 50% of the genes was below the detection limit were excluded from further analysis. In the final gene expression dataset, 100 baseline biopsies and 33 re-biopsies remained for the analysis (29 matched biopsies, 71 single baseline biopsies, 4 single re-biopsies) (Fig. 1 and Table S1).

### Impact of chemotherapy on the gene expression profile

**Fig. 2:**
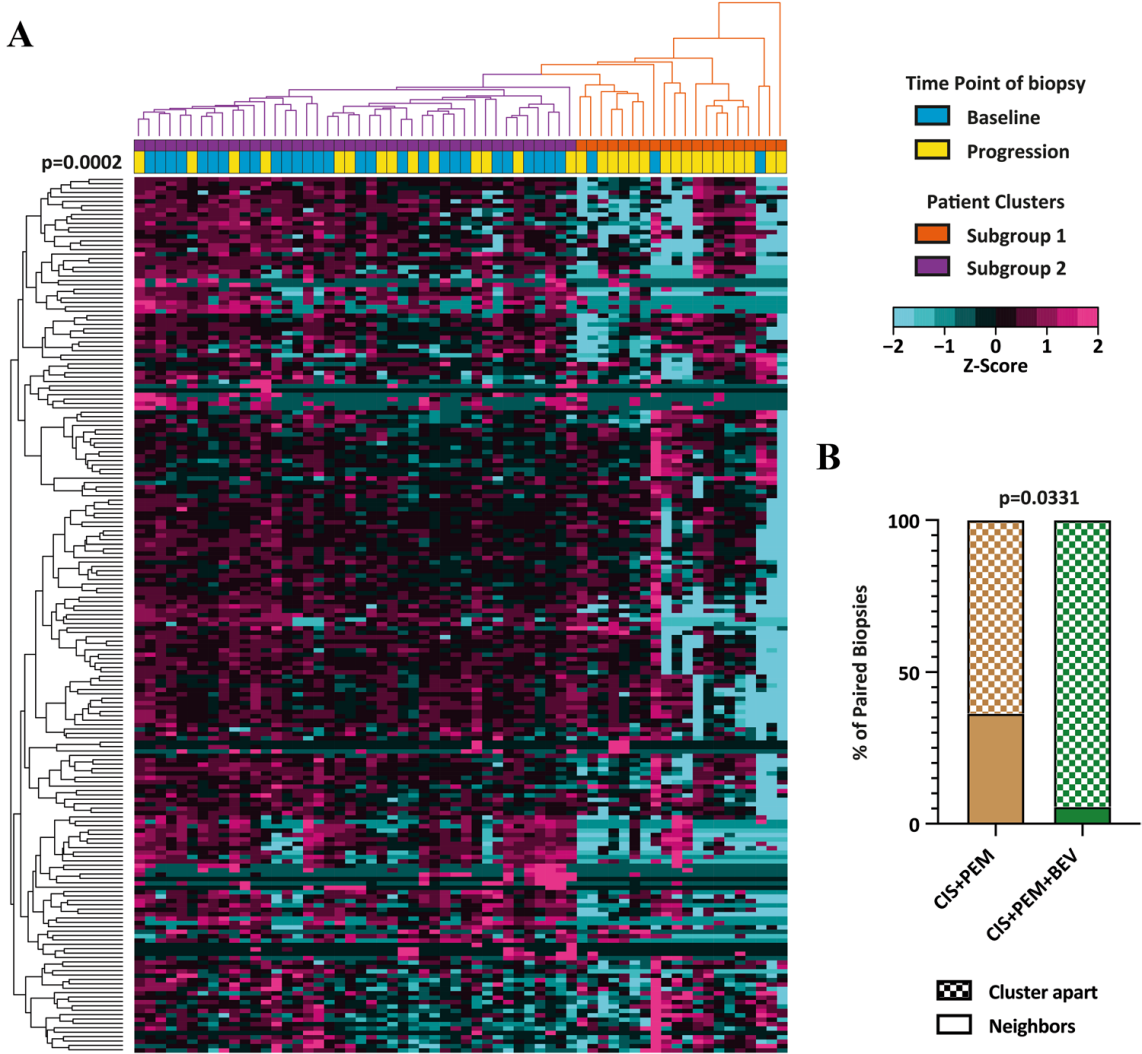
Clustering of baseline biopsies and matched rebiopsies from 29 EGFR wild type NSCLC patients. **a** Heatmap for the expression of 201 immune and cancer-related genes. Columns represent NSCLC biopsies and rows represent genes. Expression values have been centred and scaled for each row for better visualization. Rows and columns have been grouped using unsupervised hierarchal clustering. Two patient subgroups were derived from the clustering and are indicated above the heatmap: (violet) subgroup 1 (*n* = 38), (orange) subgroup 2 (*n* = 20). Blue and yellow bars indicate baseline biopsies and rebiopsies, respectively. **b** Frequency of paired pre- and post-therapy samples found to cluster together as neighbours or apart after treatment with CIS + PEM or CIS + PEM + BEV. Statistics: McNemar test was used to calculate the significance of matched samples to cluster apart from each other into subgroup 1 and subgroup 2. Chi-square test was used to calculate cluster as neighbours or apart from each other

To visualize the effect of platinum-based chemotherapy on gene expression in NSCLC, we performed unsupervised hierarchical clustering on centred and scaled gene expression levels. Clustering of a subset of patient-matched baseline/progression biopsies (*n* = 29 pairs) resulted in a separation of baseline and progression biopsies into separate clusters (*p* = 0.0002, McNemar test) (Fig. 2a). The same picture was obtained when we repeated the unsupervised hierarchical clustering on the entire dataset containing all 100 baseline biopsies and 33 rebiopsies (*n* = 133) (*p* = 0.0003, Chi-square test) (Figure S1).

With the exception of 5 paired pre- and post-treatment samples clustering right next to each other (17.2%), the remaining paired samples clustered relatively far apart. Out of these five patients, four were treated with CIS + PEM and one received CIS + PEM + BEV (Figure S2). Thus, 36.4% of CIS + PEM pre- and post-treatment samples compared to 5.6% of CIS + PEM + BEV pre- and post-treatment samples clustered next to each other (*p* = 0.0331, Chi-square test) (Figs. 2b and S2). This means that the gene expression profiles of patients receiving CIS + PEM showed a higher proportion of patient samples with high similarities between baseline and rebiopsy compared to patients receiving CIS + PEM + BEV (Fig. 2b). This suggests that the addition of BEV could increase changes in gene expression induced by chemotherapy.

### Identification of an immune gene co-expression network

**Fig. 3:**
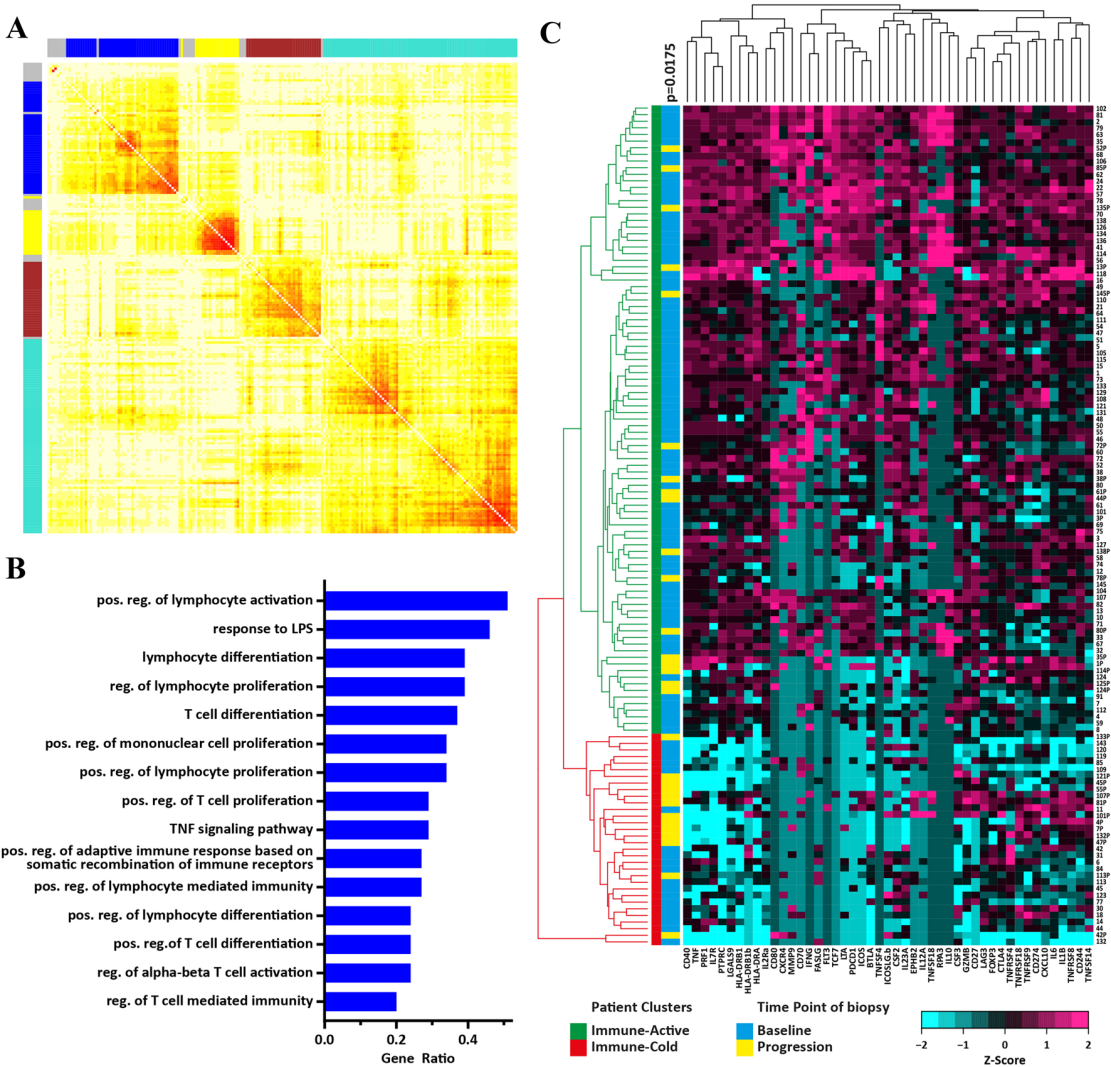
Identification of an immune module in NSCLC. **a** The entire dataset consisting of 201 genes was clustered based on a weighted gene co-expression network as represented by the correlation heatmap. The intensity of red in the heatmap represents the correlation strength between any two genes on a linear scale. The 4 identified co-expression clusters, referred to as modules, are indicated by the colors yellow, brown, blue and turquoise. **b** Barplot representing the top 15 enriched gene ontology biological process terms (*p* < 0.01) of all genes included in the blue module. **c** Unsupervised hierarchical clustering of the entire dataset (*n* = 133) based on the gene expression of genes included in the blue module(*n* = 47). Rows represent NSCLC biopsies and columns represent genes. Two patient subgroups were derived from the clustering and are indicated above the heatmap:(green) immune-active (*n* = 101), (red) immune-cold (*n* = 32). Blue and yellow bars indicate baseline biopsies and biopsies upon progression, respectively. Statistics: Chi-square test was used to calculate the significance of baseline biopsies and rebiopsies clustering apart from each other into the immune-active and immune-cold cluster

Overall differential expression analysis did not detect significantly differentially expressed genes. We attribute this to our biased and limited selection of genes and an overall heterogenous sample population. Therefore, we decided to use an unbiased approach to identify global gene co-expression networks and correlate them with the time point of biopsy. To achieve this, the expression data from the entire gene expression dataset (*n* = 133) was used to identify gene co-expression networks. The WGCNA R package was used to identify clusters of co-expressed genes, so-called modules based on average linkage hierarchical clustering [20, 21]. A power of beta = 8 was applied as the soft threshold to ensure a scale-free network. With this method, the algorithm identified 4 co-expression modules (Fig. 3a and Table S2). Next, we analyzed whether these modules correspond to specific biological functions using GO enrichment analysis. Only the blue and yellow modules enriched significantly for GO biological processes (Fig. 3a). The yellow module was enriched for genes involved in cell cycle regulation (Figure S3). The top 15 enriched GO terms in the blue module were all immune-related processes, relevant for activation, differentiation and proliferation of lymphocytes, especially T-cells (Fig. 3b). Among the 47 genes which make up the blue “immune” module we found the immune checkpoint genes PDCD1, CD274 and CTLA4.

Based on the differential expression profile of the 47 immune-related genes that constitute the blue module, we repeated an unsupervised average linkage hierarchical clustering on all samples of the dataset (*n* = 133) (Fig. 3c). This analysis divided the patients in two main clusters: (1) a cluster with low gene expression levels (immune-cold, marked by the red ribbon) and (2) a cluster with intermediate to high gene expression levels (immune-active, marked by the green ribbon). Interestingly, the immune-cold cluster was significantly enriched for biopsies collected at the time point of progression (*p* = 0.0175, Chi-square test). We observed the same result, when the analysis was restricted to the patient-matched samples (*n* = 29 pairs). The samples clustered into an immune-hot and an immune-cold cluster and re-biopsies after chemotherapy cluster significantly into the immune cold cluster (*p* = 0.0215, McNemar test) (Data not shown).

### Chemotherapy reduces the expression of immune-related genes

**Fig. 4:**
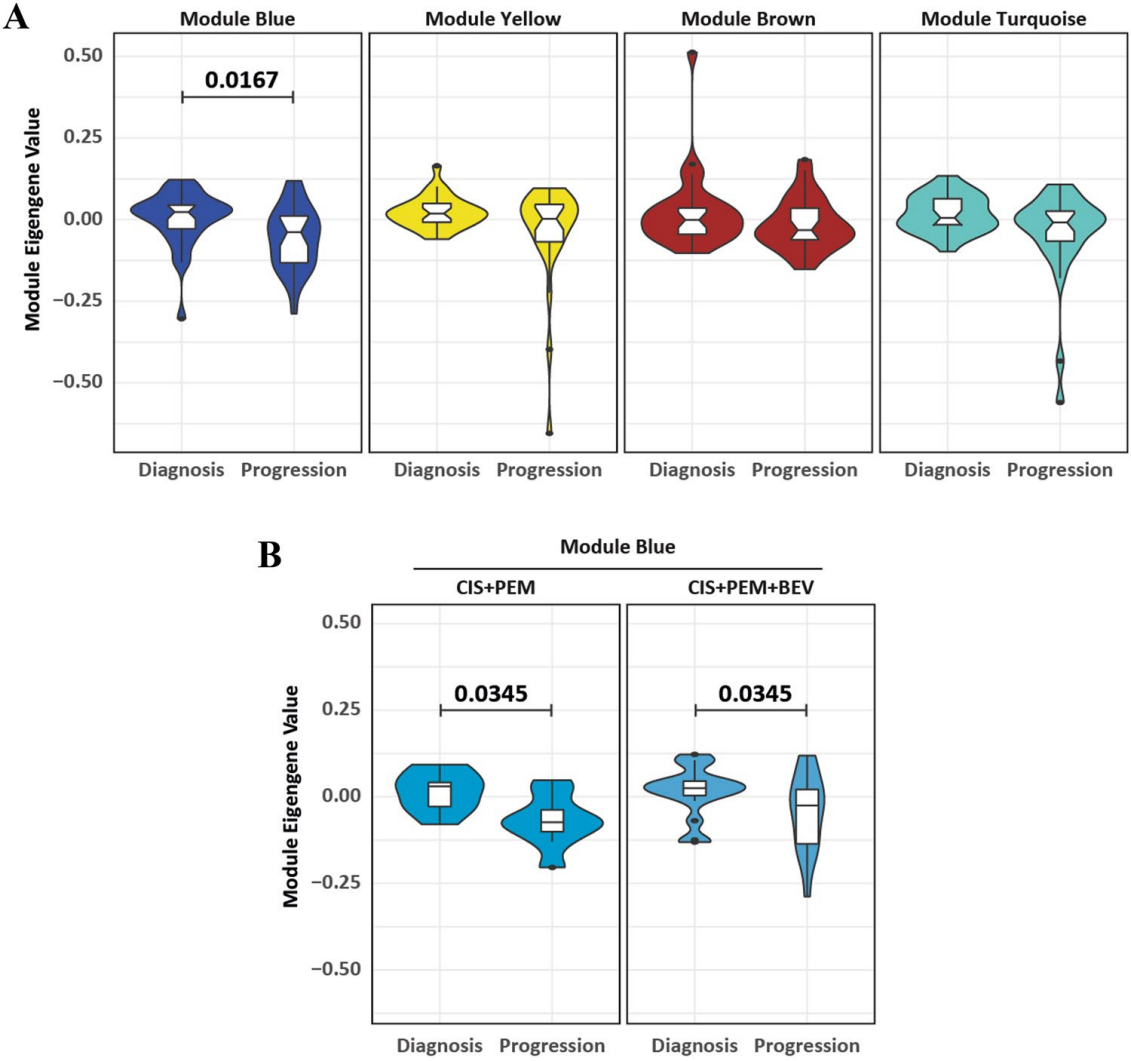
Effect of chemotherapy on co-expression modules in patient-matched biopsies. Collective expression (represented by the module eigengene) of **a** the 4 identified co-expression modules stratified by the biopsy time point and **b** the blue co-expression module further stratified by treatment arm. Statistics: Paired *T* test with Holm-Sidak for multiple comparison correction

Based on the observation that patient-matched samples at baseline and progression tended to cluster distant from each other (Fig. 2b), we compared the expression of the genes in the immune module (blue) of patient-matched samples before and after chemotherapy (*n* = 29 pairs). The expression of genes in each module was summarized in a module eigengene (ME) expression value based on the first principal component of this module, which can be considered as the best summary of the standardized expression data of the genes included in the module. We then compared MEs between all paired baseline/progression samples. We found that the blue immune module was the only one to be significantly reduced after chemotherapy (*p* = 0.0167; Paired *T* test with Holm Sidak correction) (Fig. 4a). This is in line with the observation that the re-biopsies clustered at a higher frequency in the immune cold cluster (*p* = 0.0175, Chi-square test) (Fig. 3c). This reduction of immune-related genes was observed in both cohorts treated either with CIS + PEM + BEV (*p* = 0.0345; Paired *T* test with Holm Sidak correction) or with CIS + PEM (*p* = 0.0345; Paired *T* test with Holm Sidak correction) (Fig. 4b).

### Chemotherapy causes down regulation of CTLA4, FOXP3, LAG3, TNFRSF18 and CD80

**Fig. 5:**
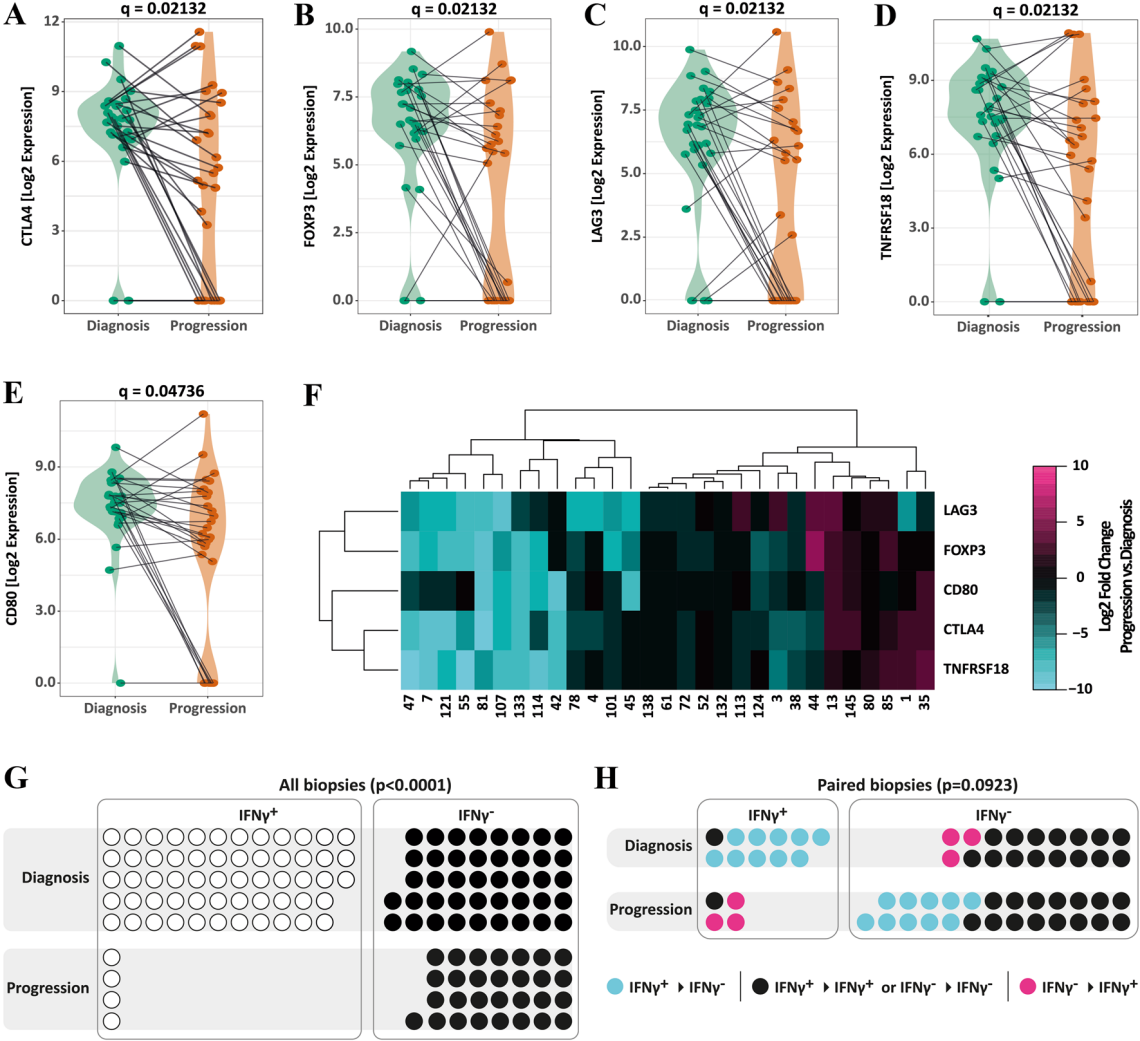
Differently expressed blue module genes in patient-matched biopsies. Differential gene expression of genes belonging to the blue module (*n* = 47) was analyzed in patient-matched baseline/progression biopsies (*n* = 29 pairs). Genes found to be significantly differently expressed were **a** CTLA4, **b** FOXP3, **c** LAG3, **c** TNFRSF18 and **b** CD80. Statistics: Paired t-test with FDR (*q* = 0.05) for multiple comparison correction. **f** Heatmap representing the changes in gene expression of CTLA4, FOXP3, LAG3, TNFRSF18 and CD80 between the rebiopsy and baseline biopsy (Log2 fold change). Rows and columns have been grouped using unsupervised hierarchal clustering. **g**, **h** IFNγ expression status (IFNγ^+^ or IFNγ^−^) at baseline and progression of **g** all biopsies and **h** only paired Biopsies. Each circle represents a patient. For paired samples yellow circles represent patients without a change in the IFNγ expression status, orange circles represent IFNγ^+^ patients which become IFNγ^−^ and blue circles represent IFNγ^−^ patients which become IFNγ^+^. Statistics: Chi-square test (**g**) and McNemar test (**h**)

As chemotherapy only had an impact on the overall expression of the immune module (Fig. 4), we restricted the differential gene expression analysis to the latter. Of the 47 immune-related genes analyzed in 58 patient-matched baseline/progression samples (*n* = 29 pairs), we found 5 genes to be significantly reduced after chemotherapy (paired *T* test with controlling false discovery rate (FDR) at *q* = 0.05). Genes that were significantly reduced in re-biopsies included cytotoxic T-lymphocyte-associated protein 4 (CTLA4; *p* = 0.00141; *q* = 0.02132), Forkhead-Box-Protein P3 (FOXP3; *p* = 0.00147; *q* = 0.02132), lymphocyte-activation gene 3 (LAG3; *p* = 0.00177; *q* = 0.02132), tumor necrosis factor receptor superfamily member 18 (TNFRSF18; *p* = 0.00181; *q* = 0.02132), and cluster of differentiation 80 (CD80; *p* = 0.05038; *q* = 0.04736) (Fig. 5a–e). Down-regulation of one of these genes was accompanied by the down-regulation of several or all of the five immune-related genes (Fig. 5f). Next, we tested whether the observed reduction of the average expression of the immune module was mainly the result of the changes in the expression of CTLA4, FOXP3. LAG3, TNFRSF18 and CD80. Therefore, we calculated the blue module eigengene values without including CTLA4, FOXP3, LAG3, TNFRSF18 and CD80. Despite removing these genes the blue module eigengene was still significantly reduced after treatment with chemotherapy (Figure S4). This indicates that the observed reduced expression of a panel of immune-related genes in the blue module did not depend on a few defined genes.

Even though interferon-γ (IFN-γ) was not significantly differently expressed in matched patient samples (*p* = 0.1774, paired t-test with controlling FDR at *q* = 0.05), we included this cytokine in the analysis based on a recent publication by Higgs et al. [23] They found that survival of NSCLC patients receiving durvalumab correlated with detectable IFN-γ mRNA in tumor biopsies. Overall, we detected IFN-γ in 58 out of 100 baseline samples (58%) and in 4 out of 33 progression samples (12.1%) (Fig. 5g). Restricting the analysis to matched patient samples, we detected mRNA expression in 11 out of 29 baseline biopsies (34.5%). After chemotherapy, IFN-γ expression was detectable only in 4 out of the 29 patient-matched re-biopsies (13.8%). Interestingly, only 1 out of the 11 IFN-γ^+^ baseline samples remained IFN-γ^+^ after progression (Fig. 5h). While binominal analysis of IFN-γ expression is significantly reduced in the rebiopsies (*p* < 0.0001, Chi-square test) over all samples, we only observed a trend in patient-matched samples (*p* = 0.0923, McNemar test).

## Discussion

It has become increasingly clear that the success of immune checkpoint inhibition is strongly linked to the presence of a pre-existing T cell response against the tumor [23]. Several studies have analyzed the immune tumor microenvironment by gene expression profiling in the search of predictive bio-markers. A retrospective analysis of 50 tumor samples from melanoma patients treated with the anti-CTLA4 antibody ipilimumab provided the first evidence that gene expression profiling could be useful as a predictive biomarker [15]. Clinical activity (objective response or stable disease ≥ 24 weeks) with ipilimumab correlated with an active immune microenvironment in the tumor. The expression of 22 immune-related genes including the cytotoxic T cell markers granzyme B (GZMB), perforin (PRF1), as well as MHC class II HLA-DQA1 correlated with response to immunotherapy [15]. Ribas et al. presented two IFN-γ-related gene signatures with predictive value, a 10-gene and an expanded 28-gene immune signature, including IFNG, CXCL10, LAG3, GZMB and HLA-DR [24].

In the present analysis, we identified a module of 47 immune-related genes that were co-expressed in non-squamous NSCLC EGFR wild-type tumors. The module consisted of genes involved in the regulation of the adaptive immune response similar to the immune profiles defined by the two other groups [15, 24]. Standard platinum-based chemotherapy had a negative impact on the expression of this immune gene module. Importantly, chemotherapy reduced the average expression of a panel of immune-related genes, even though the overall differential expression analysis did not detect a differential expression in a single defined gene. Although CTLA4, TNFRSF18, LAG3, CD80 and FOXP3 gene expression were found to be significantly reduced when the analysis was limited to the immune module, these defined genes did not determine the overall effect of chemotherapy on the immune module.

Even though BEV has been reported to have positive immune-modulatory effects we did not observe any major differences in chemotherapy-induced changes of the immune microenvironment between CIS + PEM and CIS + PEM + BEV treated patients [25]. In contrast, the IMpower150 trial recently reported improved overall survival (OS) with first-line atezolizumab in combination with carboplatin, paclitaxel and bevacizumab over carboplatin, paclitaxel and bevacizumab. Interestingly, atezolizumab in combination with carboplatin and paclitaxel alone did not improve OS [26, 27]. Our data suggest that BEV might act synergistically with immune checkpoint blockade via other mechanisms than maintaining or supporting the immune infiltrate in the tumor.

Higs et al. demonstrated that NSCLC patients with detectable IFN-γ mRNA expression in the tumor responded better to durvalumab compared to patients with no detectable levels of IFN-γ [28]. In our study, over the course of chemotherapy until progression, only one out of ten patients remained IFN- positive and nine lost IFN-γ expression, while four previously IFN-negative patients turned IFN-positive. This suggests that IFN-γ mRNA expression in the tumor should be analysed directly before immunotherapy and not in archival samples taken at the time point of diagnosis before chemotherapy.

The expression of CTLA4, TNFRSF18, LAG 3and CD80 and the regulatory T-cell marker FOXP3 were significantly reduced when analysed as single genes in the biopsy samples taken at progression after first-line chemotherapy and when the statistical analysis was limited to the immune module. CTLA4 and PD1 are both expressed on activated T cells. CTLA-4 inhibits early T cell activation and cell cycle progression, whereas PD-1 primarily inhibits T cell function in the effector phase [29]. The anti-CTLA4 antibody ipilimumab was the first immune checkpoint inhibitor to get FDA approval. However, single-agent immune checkpoint blockade with anti-CTLA4 antibodies have not been efficacious in patients with metastatic NSCLC [30]. Currently, multiple clinical trials investigate the therapeutic potential of combined CTLA4/PD-1 blockade in NSCLC patients (NCT03409614, NCT02453282, NCT03319316). Besides PD1 and CTLA4, LAG3 is another important immune checkpoint receptor that was found to be expressed on activated TILs but not NSCLC cells [31]. Preclinical studies have found that inhibition of LAG3 allowed cytotoxic T-cells to regain cytotoxic function similar to PD-1/PD-L1 inhibition [32]. A phase I/IIa study is currently testing an anti-LAG3 antibody (BMS-986016) in immune therapy refractory solid tumors (NCT01968109). Multiple preclinical studies have provided evidence that stimulation of GITR (TNFRSF18) in the tumor microenvironment contributes to costimulatory activation of CD4 and CD8 T-cells, while inhibiting/depleting intra-tumoral regulatory T cells [33].

To our knowledge, our study is the first analysis of the immune microenvironment in metastatic non-squamous NSCLC before and after standard platinum-based chemotherapy. Despite the relatively low number of samples, we document that the average expression of a panel of 47 immune-related co-expressed genes is significantly down-regulated after platinum-based chemotherapy. Altogether our data suggests that conventional platinum-based chemotherapy negatively impacts anit-tumoral immunity.

### Electronic supplementary material

Below is the link to the electronic supplementary material.

Supplementary file1 (PDF 905 kb)
