# Supplementary material for: Chemotherapy negatively impacts the tumor immune microenvironment in NSCLC: an analysis of pre- and post-treatment biopsies in the multi-center SAKK19/09 study
Source: Cancer Immunol Immunother. 2020 Aug 7;70(2):405–15. doi: 10.1007/s00262-020-02688-4 (PMC7889678; doi:10.1007/s00262-020-02688-4)
Supplement: Supplementary file 1 — Supplementary file1 (PDF 905 kb) [file 262_2020_2688_MOESM1_ESM.pdf]

Supplementary Materials

Table S1

Table S1: Coverage of tumor sampling

	Overall (n=129)		CIS+PEM+BEV (n=77)		CIS+PEM (n=52)	
	n	%	n	%	n	%
Baseline biopsies	123	95.3	74	96.1	49	94.2
NanoString analysis	116	89.9	69	89.6	47	90.4
Good read quality	100	77.5	65	84.4	25	48.0
Rebiopsies	62	48.1	36	46.8	26	50.0
NanoString analysis	36	27.4	22	28.6	14	26.9
Good read quality	33	25.6	20	26.0	13	25.0

Table S2

Table S2: WGCNA Modules

Blue Module	Yellow Module	Brown Module	Turquoise Module
BTLA, CD244, CD27, CD274, CD40, CD70, CD80, CSF2, CSF3, CTLA4, CXCL10, CXCR4, EPHB2, FASLG, FLT3, FOXP3, GZMB, HLADRA, HLADRB1, HLADRB1b, ICOS, ICOSLGb, IFNG, IL10, IL12A, IL1B, IL23A, IL2Ra, IL6, IL7R, LAG3, LGALS9, LTA, MMP9, PDCD1, PRF1, PTPRC, RPA3, TCF7, TNF, TNFRSF18, TNFRSF4, TNFRSF8, TNFRSF9, TNFSF14, TNFSF18, TNFSF4	ANGPTL4, AURKA, AURKB, BIRC5, BRCA1, BRCA2, BRIP1, CCNB2, CCNE1, CDC20, CDC25C, CDC6, CDK1, CDK2, MYC, PMS2, POLD3, PTGS, TGFA, TYMS	AKT1, ATR, BAX, BCL2, CDC34, CDKN1B, DDB1, ERCC1, ERCC3, ERCC5, ERCC8, IKBKB, MLH1, MSH5, MSI2, NFKB2, NOTCH1, NRP1, NRP2, Numb, PLCG1, PNKP, RUNX1, RUNX2, STAT3, TGFB1, TGFBR1, TNFRSF14, TNIK, TRAF2, TRAF5, TREX1	ANG, ANGPT1, ANGPT2, ANGPTL1, ANGPTL2, ATM, CCNH, CD86, CDC16, CDK4, CDK6, CDK7, CDK8, CDKN1A, CDKN2B, CEBPA, CSF1, CTNNB1, CXCL12, DDB2, ERCC2, ERCC4, ERCC6, FAS, FGF1, FGF2, FGFR3, FLT1, FLT4, HAVCR2, HGF, ICOSLG, IL33, JUN, LEF1, LTBR, MAPK1, MDM2, MLH3, MMP2, MSH2, MSH3, MSH6, MSI1, MTHFR, NFKB1, PDCD1LG2, PDGFA, PDGFB, PDGFRA, PDGFRB, PECAM1, PIK3CA, PLXDC1, PMS1, POLL, RAD23A, RAD23B, RPA1, SFRP2, SLK, SMO, SP1, ST2, TCF4, TGFB2, TGFB3, TIMP1, TIMP2, TIMP3, TNFRSF19, TP53, TP73, VEGFB, VEGFC, WISP1, WISP2, WNT5A, WNT5B, WNT9A, XAB2, XPA, XPC

Figure S1

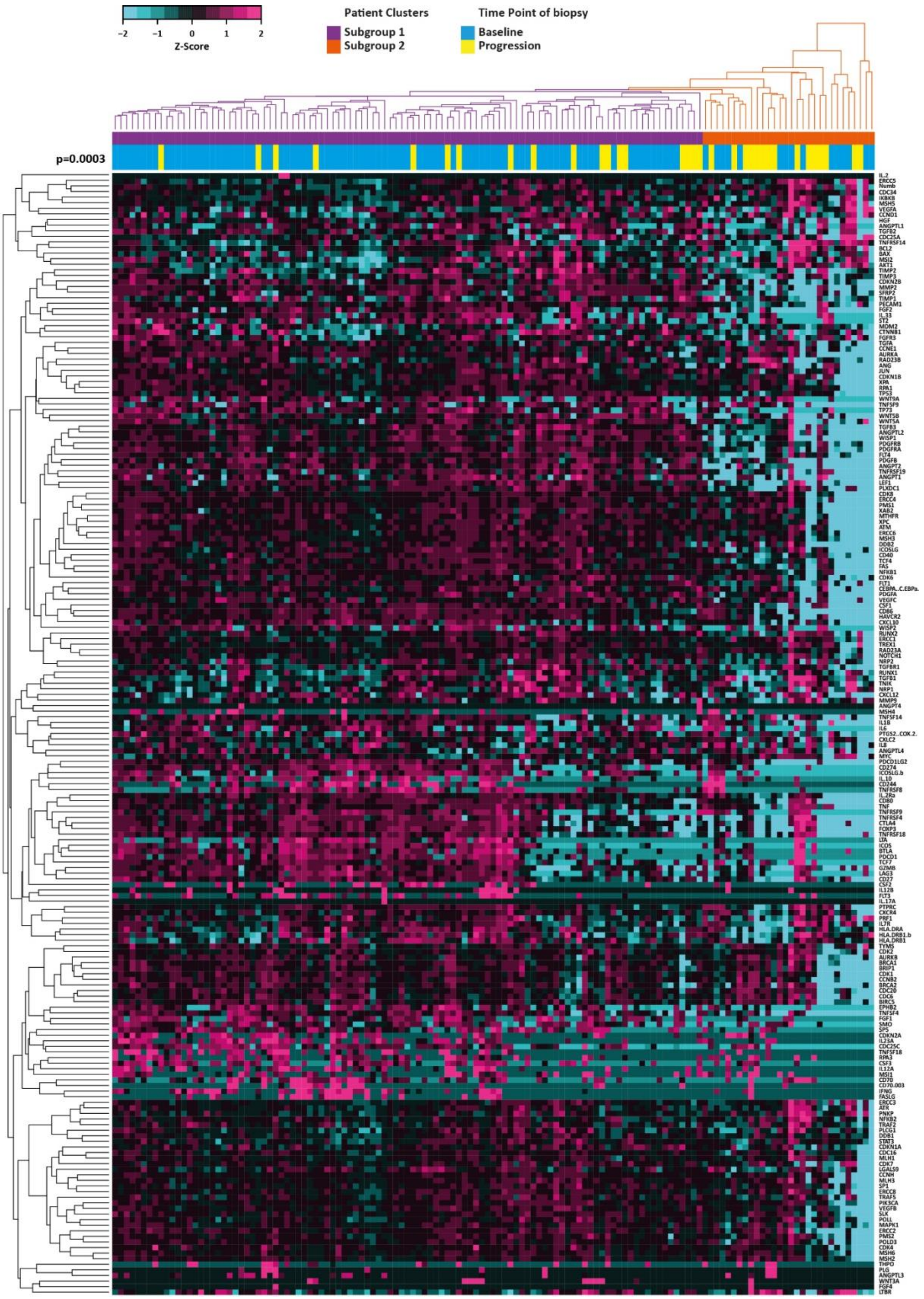
-2 -1 0 1 2
Z-Score
Patient Clusters
Subgroup 1
Subgroup 2
Time Point of biopsy
Baseline
Progression
p=0.0003

Figure S1

Gene expression pattern of all analyzed NSCLC biopsies. Heatmap for the expression of 201 immune and cancer related genes. Columns represent NSCLC biopsies and rows represent genes. Expression values have been centred and scaled for each row for better visualization. Rows and columns have been grouped using unsupervised hierarchical clustering. Two patient subgroups were derived from the clustering and are indicate above the heatmap: subgroup 1 (n = 103, violet) subgroup 2 (n = 30, orange). Blue and yellow bars indicate baseline biopsies and rebiopsies, respectively.

Figure S2

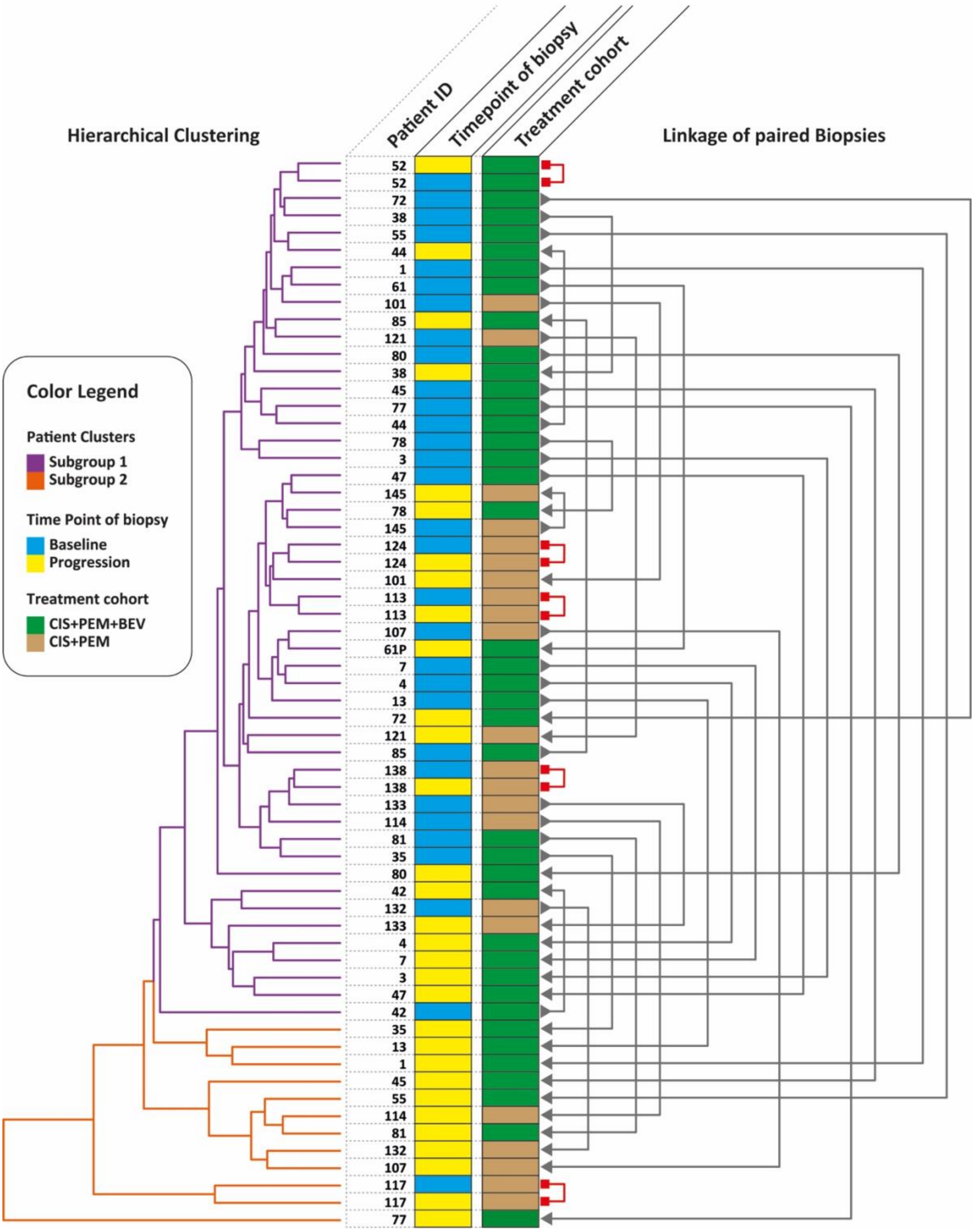
Hierarchical Clustering
Patient ID
Timepoint of biopsy
Treatment cohort
Linkage of paired Biopsies
Color Legend
Patient Clusters
Subgroup 1
Subgroup 2
Time Point of biopsy
Baseline
Progression
Treatment cohort
CIS+PEM+BEV
CIS+PEM
52
52
72
38
55
44
1
61
101
85
121
80
38
45
77
44
78
3
47
145
78
145
124
124
101
113
113
107
61P
7
4
13
72
121
85
138
138
133
114
81
35
80
42
132
133
4
7
3
47
42
35
13
1
45
55
114
81
132
107
117
117
77

Figure S2

Clustering of baseline biopsies and matched rebiopsies from 29 EGFR wild type NSCLC patients. Dendrogram showing patient clustering. Tiled columns indicate patient id, time point of biopsy and treatment cohort. Connecting lines on the right indicate matched pairs. Grey lines indicate matched biopsies clustering apart; red lines indicate matched biopsies clustering next to each other.

Figure S3

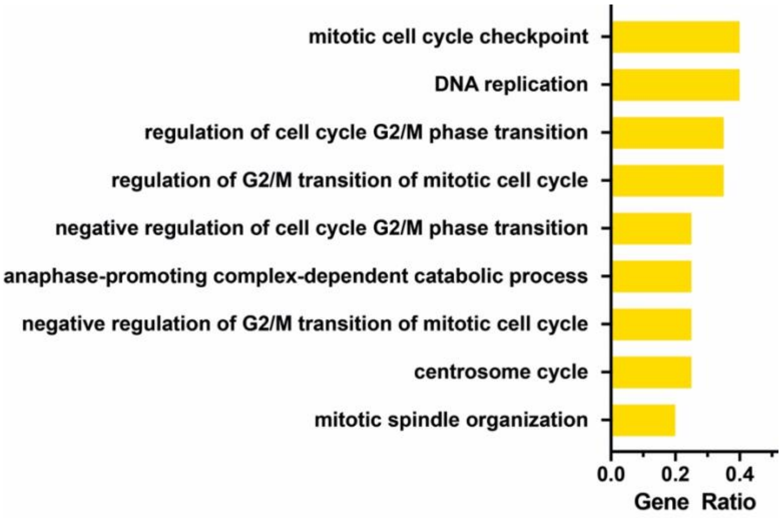

Yellow module is enriched for genes involved in cell cycle regulation. Barplot representing the 9 enriched gene ontology biological process terms (p<0.01) of all genes included in the yellow module.

Figure S4

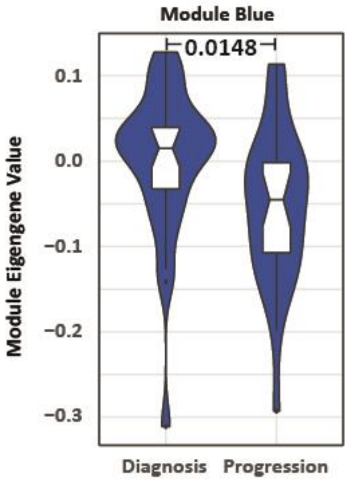

The decreased expression of the immune module is not dependent on CTLA4, LAG3, CD80, FOXP3 and TNFRSF18. The module eigengenes (collective expression) for the blue module were calculated without including CTLA4, LAG3, CD80, FOXP3 and TNFRSF18. The module eigengene values are plotted for paired patient-samples at timepoint of diagnosis and progression. Statistics: Paired T-test.
